# Actinomycosis of Cecum Associated with *Entamoeba* Infection Mimicking Perforated Colon Cancer

**DOI:** 10.1155/2013/143218

**Published:** 2013-04-30

**Authors:** Deniz Eren Böler, Cihan Uras, Süha Göksel, Mehmet Karaarslan

**Affiliations:** ^1^Department of General Surgery, Acibadem University Medical Faculty, Acibadem Bakirköy Hospital, Halit Ziya Uşakligil Caddesi No. 1 Bakirköy, 34140 Istanbul, Turkey; ^2^Department of Pathology, Acibadem Maslak Hospital, 34457 Istanbul, Turkey; ^3^Department of Internal Medicine, Acibadem University Medical Faculty, 34848 Istanbul, Turkey

## Abstract

Actinomycosis is a granulomatous disease caused by *Actinomyces* that mimics other intra-abdominal pathologies especially neoplasms. Correct diagnosis can be rarely established before radical surgery. On the other hand *Entamoeba* infection affects a considerable number of people worldwide. To our knowledge only one case has been reported to be affected by both organisms. We report a man who has been operated for a mass in the cecum mimicking a perforated colon cancer. Abdominal CT revealed a mass with features of an invading neoplasm. After radical surgery, definitive pathology revealed that the mass was due to actinomycosis associated with *Entamoeba* infection. The postoperative period was uneventful and the patient was on long-course antibiotherapy. It is important to consider actinomycosis especially in patients with intra-abdominal masses with unusual aggressiveness to prevent unnecessary surgery. However, surgery can be unavoidable especially in the presence of complicated disease or high index of suspicion for malignancy.

## 1. Introduction

*Actinomyces* is an anaerobic, gram-positive saprophytic organism normally present in the gastrointestinal tract, female genital tract, and bronchus [1]. It is not always pathologic but it may lead to chronic infectious diseases with destruction of muscular barrier by trauma, endoscopic manipulations, previous operations, gastrointestinal foreign body, and infections like appendicitis [1–3]. The infection is facilitated by immunosuppressive conditions like leukemia, lymphoma, renal transplant, and diabetes [4]. Bowel obstruction and perforation without predisposing factors are very rare and only a few cases have been described in the literature [3]. The clinical course is indolent and a malignant tumor-like appearance makes differential diagnosis difficult that leads to a delay in treatment [1]. 

On the other hand, *Entamoeba* infections are prevalent worldwide and the clinical course may vary from asymptomatic states to “amebomas” which are exophytic, cicatricial, and inflammatory masses due to longstanding and partially treated infections. These are seen in only 1.5% of patients with amebiasis [5]. The differentiation of these masses from Crohn's disease, abscesses due to perforated appendicitis, colon cancer, and diverticulosis is important for early diagnosis and treatment [5, 6]. 

To our knowledge the presence of *Entamoeba* in a mass formed by* Actinomyces* infection has not been reported in the literature and the only report regarding the association of these two microorganisms is by Arroyo who wrote about an intrauterine contraceptive device user colonized by *Actinomyces* and *Entamoeba* [7]. 

The present paper discusses a case of actinomycosis associated with* Entamoeba* leading to a mass mistaken for perforated colonic carcinoma in a 52-year-old man.

## 2. Case Presentation

**Figure 1:**
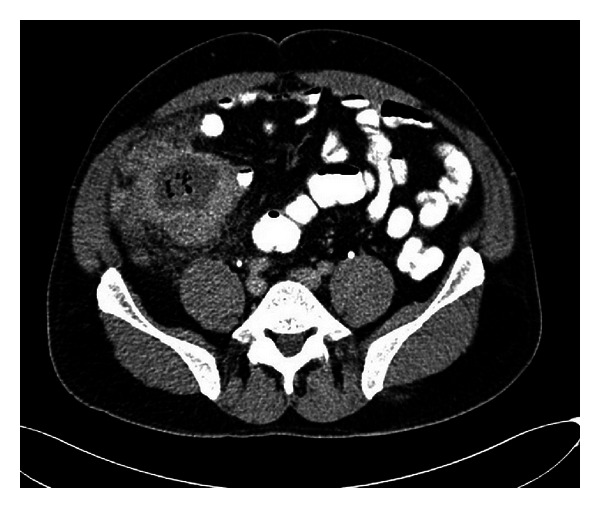
CT image of the mass located in the cecum.

A 52-year-old man applied to the outpatient clinics with the complaints of abdominal pain and weight loss for two months. Abdominal pain had worsened for the last two days. He also complained of diarrhea for the last week. His past history revealed restless leg syndrome. He had undergone an operation for sinusitis four months before the admittance day. Physical examination revealed a mass and tenderness in the right lower quadrant with local signs of peritonitis. White blood cell count was 11,330/mm^3^; haemoglobin level was 11 gr/dL with normal platelet count. Liver and kidney function tests were in normal limits except an increased blood glucose level (146 gr/dL) but the patient denied a history of diabetes. Computerized tomography (CT) scans of the abdomen demonstrated a 90 × 83 × 95 mm mass with gas containing abscess involving cecum and distal ileum with luminal narrowing and marked inflammatory changes in the contiguous tissues. Multiple lymph nodes measuring up to 19 × 11 mm were seen in the pericecal region (Figure 1). On the same day, the patient was referred to the general surgery department with suspicion of perforated colonic carcinoma. Colonoscopy was not performed. Broad spectrum antibiotics were given and the patient underwent laparotomy and right hemicolectomy with segmental ileal resection and partial omentectomy. The intraoperative findings were compatible with perforated cecal neoplasm that invaded the parietal peritoneum. The postoperative period was uneventful and the patient was discharged on postoperative day 9.

**Figure 2:**
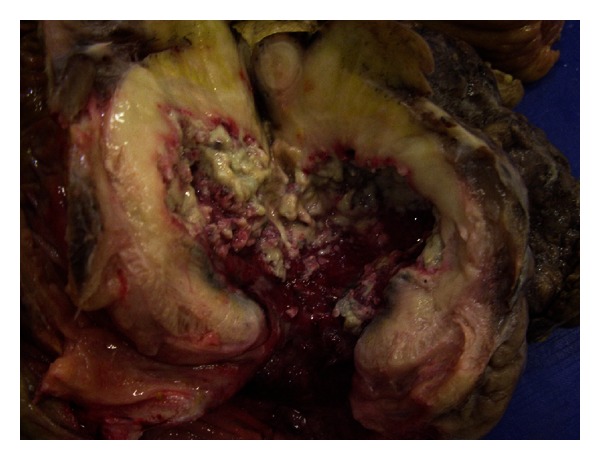
Macroscopic appearance of the specimen and sulfur granules in the cavity.

**Figure 3:**
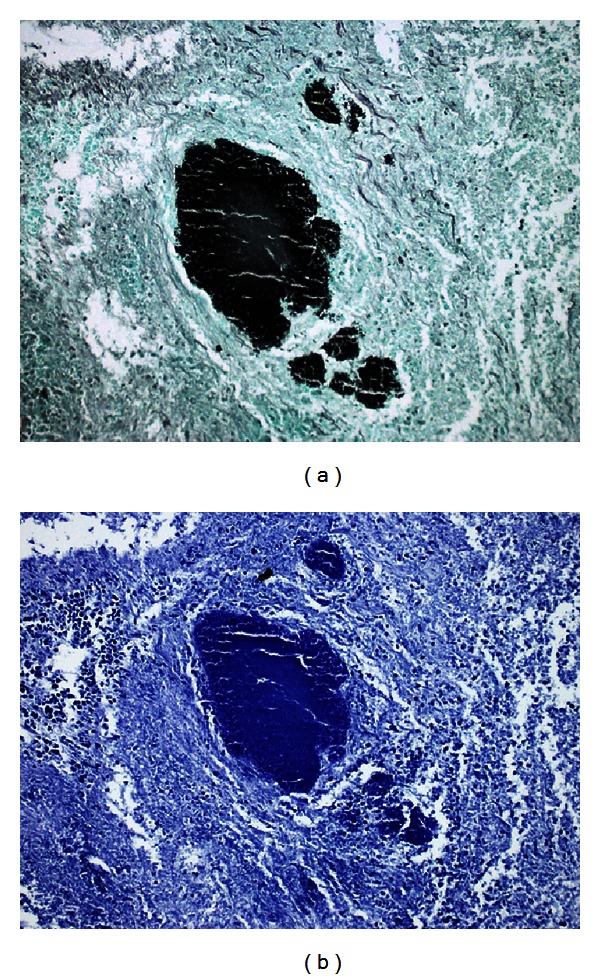
Microscopic appearance of colonies of *Actinomyces* with periodic acid-Schiff and Grocott's dye (×40).

**Figure 4:**
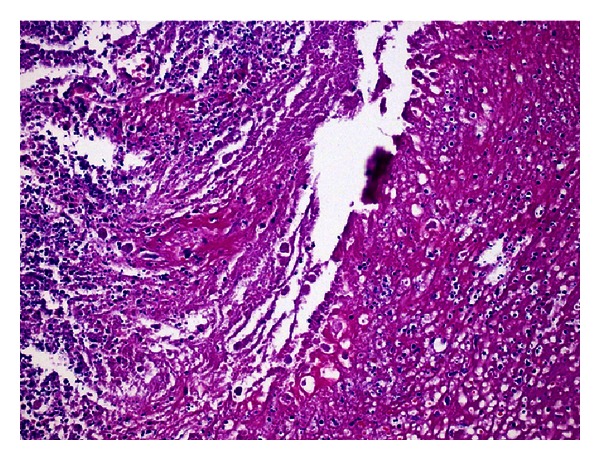
Microscopic appearance of *Entamoeba* trophozoites on the surface of necrotic tissue (H.E. ×40).

The mass was 11 × 8 × 3 cm in macroscopic evaluation. Pathology revealed pseudotumor formation with necrosis of cecum and ileocecal valve (Figure 2) involving multifocal colonies of *Actinomyces* with periodic acid-Schiff and Grocott's dye (Figure 3). There were multifocal *Entamoeba* trophozoites on the surface of the necrotic tissue (Figure 4). Transmural and mesenteric fibrosis with lymphocytic infiltrate was seen. Similar inflammatory granulomatous process was present in the terminal ileum and adjacent structures including appendix.

## 3. Discussion

Actinomycosis is a chronic suppurative disease characterized by the formation of multiple abscesses, draining sinuses, abundant granulation, and dense fibrous tissue [8]. Actinomycosis of the abdomen and pelvis accounts for 10%–20% of the reported cases [9] and ileocecal region and appendix are the most commonly involved regions [1]. About 80% of pelvic actinomycosis has been reported in women using intrauterine device for more than four years [10, 11]. Appendicitis, diverticulitis, inflammatory bowel disease, and previous surgery are other causes of infection [1, 12]. 

The diagnosis of abdominal actinomycosis is challenging before surgical intervention. The clinical appearance is not specific and the most common symptom is abdominal pain although it may depend on the involved organ [10, 13]. The course of the disease is indolent and it mimics other diseases like appendicitis, diverticulitis, colon carcinoma, Crohn's disease, ulcerative colitis, and tuboovarian abscess [14]. 

Computerized tomography is an important imaging modality for diagnosis, degree of involvement, and monitoring the effectiveness of the treatment [15, 16]. Direct spread into the adjacent tissue is the most common primary route of propagation although the mode of spread is not fully understood. Infiltrative mass with tendency to cross-boundaries and fascial planes involves multiple compartments and extent of the abdominal wall has been well described [16, 17]. After infusion of the contrast material dense contrast enhancement in the mass or involved bowel which may be caused by abundant granulation and dense fibrous tissue has been reported [15–18]. The aggressiveness has been noted to be unusual with absence of ascites and lymphadenomegaly [1, 16]. Because of the size of the organism, spread via lymphatic system is unlikely or develops in the late course of the disease [1, 12, 19]. In the present case there were pathological lymph nodes measuring up to 2 cm in the pericecal area. The enlarged lymph nodes may be due to longstanding disease or associated *Entamoeba* infection. The harvested lymph nodes were reported to be reactive in the final pathological examination. The order of infective process in the present case is contentious. Asymptomatic* Entamoeba* infection might have led to actinomycosis with destruction of the mucosal barrier although there has been no evidence supported by the literature. The second scenario is that the patient has been infected by* Entamoeba* after actinomycosis developed. Whatever is true, the patient has ended up by surgery which is not the primary treatment for both infections. 

The major flaw in preoperative diagnosis is that the findings in the imaging modalities cannot discriminate between actinomycosis and malignant process, Crohn's disease, appendicitis, diverticulitis, or tuberculosis [1, 20]. Fine needle aspiration biopsy has been recommended to be used to rule out actinomycosis [21] whereas others found it inconclusive due to extensive inflammatory tissue surrounding the filaments and sulfur granules of *Actinomyces* [1]. In the majority of the patients, definitive diagnosis is reached by macroscopic, microscopic, and histochemical examinations of the specimen after surgical exploration [1] whereas some have suggested that definitive diagnosis is based on tissue culture [9]. However, *Actinomyces* cultures can yield a false negative result in up to 76% of abdominal actinomycosis [8]. In the present case no tissue culture was obtained because the specimen was thought to be neoplastic. After pathological evaluation characteristic gram-positive sulfur granules with a mycellium-like structure were seen. *Actinomyces *granules regularly show a positive reaction with periodic acid-Schiff and Grocott's dye which differentiates them from *Nocardia* and *Streptomyces* species [1].

Reports increasingly support that medical therapy alone, without surgical exploration, is usually sufficient for cure, irrespective of extensive actinomycosis [13, 22]. Treatment of actinomycosis consists of intravenous penicillin-G for four weeks and then oral penicillin for 6–12 months [23, 24]. Although no true surgical intervention guidelines have been established, operative treatment has been used in patients who present with extensive necrotic tissue or large abscesses that cannot be adequately drained [25, 26]. 

Our patient received ceftriaxone and metronidazole until the definitive pathology. Afterwards he received amoxicillin plus clavulanic acid and metronidazole [24]. After one-year followup, no complication has been noted in the patient.

## 4. Conclusion

Abdominal actinomycosis can be associated with *Entamoeba* infection. Whatever the presentation is, main challenge in abdominal actinomycosis is preoperative diagnosis. Imaging modalities and tissue samples may not be conclusive and surgery may be necessary to exclude other intra-abdominal pathologies especially malignant processes. The clinician should be aware of potential pitfalls in diagnosis and treatment whereas maintaining suspicion for actinomycosis may prevent unnecessary radical surgery for presumed pelvic malignancies.
